# Women living with HIV: perception regarding diagnosis, treatment and mental health

**DOI:** 10.1590/0034-7167-2024-0134

**Published:** 2025-03-17

**Authors:** Aline de Lima Barbosa, Amuzza Aylla Pereira dos Santos, Christefany Régia Braz Costa, Tâmara Silva de Lucena, Viviane dos Santos Melo, Lea Christine Ferreira de Araújo, Davi Porfirio da Silva, Dayse Carla Alves Sales Pereira

**Affiliations:** I Universidade Federal de Alagoas Maceió Alagoas Brazil Universidade Federal de Alagoas. Maceió, Alagoas, Brazil

**Keywords:** Perception, Women, HIV, Treatment Adherence, Antiretroviral Agents, Percepción, Mujeres, HIV, Adherencia al Tratamiento, Antirretrovirales

## Abstract

**Objectives:**

to understand the perception of women living with HIV regarding their diagnosis, treatment, and mental health.

**Methods:**

this qualitative study was conducted with eight women between November and December 2022. Data were analyzed using Bardin’s Content Analysis technique, based on the participants’ narratives and aligned with Jean Watson’s Theory of Human Caring.

**Results:**

The content was organized into two categories: Feelings upon discovering the disease and its relationship to treatment; The impact of HIV/AIDS on the mental health of women and their relationship with healthcare professionals.

**Final Considerations:**

based on the participants’ narratives, it is concluded that women’s perceptions of their HIV diagnosis, treatment, and mental health are marked by moments of fragility, making them vulnerable during the process of discovering the infection, particularly as it impacts their individual and collective dynamics.

## RESUMEN

**Objetivos:**

conocer la percepción de las mujeres que viven con VIH acerca del diagnóstico, tratamiento y salud mental.

**Métodos:**

se trata de un estudio cualitativo, realizado con ocho mujeres, en el período de noviembre a diciembre de 2022. Para el análisis de los datos, se utilizó la técnica de Análisis de Contenido de Bardin, a partir de los discursos de las participantes, en conjunto con la Teoría del Cuidado Humano de Jean Watson.

**Resultados:**

el contenido se organizó en categorías, a saber: Sentimientos ante el descubrimiento de la enfermedad y su relación con el tratamiento; Impacto en la salud mental de las mujeres que viven con VIH/SIDA y su relación con los profesionales de la salud.

**Consideraciones Finales:**

se concluye, a partir de los discursos, que la percepción de las mujeres con VIH en relación con el diagnóstico, tratamiento y la salud mental está marcada por un momento de fragilidad, lo que las hace vulnerables en el proceso de descubrimiento de la infección, principalmente por el impacto que tiene en toda su dinámica individual y colectiva.

## INTRODUCTION

The Acquired Immunodeficiency Syndrome (AIDS; SIDA in Portuguese – Acquired Immunodeficiency Syndrome) was identified in the 1970s in the United States of America (USA). It is a chronic condition associated with infection by the Human Immunodeficiency Virus (HIV) and represents a global public health issue, compromising the immune system, which is responsible for defending the body against diseases^(1,2)^.

With the advancement of science and the introduction of antiretroviral therapy (ART), the reality of people living with HIV (PLHIV) has changed, resulting in a reduction in morbidity and mortality. This has provided individuals infected with the virus not only an increased life expectancy and survival rate but also an improved quality of life, enabling the reconstruction of personal goals, including romantic relationships and the possibility of starting a family^(3)^.

In this context, early diagnosis of HIV is aimed at reducing morbidity and mortality, but most importantly, at supporting the reconstruction of personal goals for PLHIV who have not yet identified the disease. Early diagnosis allows for timely treatment and appropriate follow-up^(4)^. Thus, to provide greater care opportunities for PLHIV, the Brazilian Unified Health System (SUS) established Specialized Care Services (SAE in Portuguese) in the 1980s^(5,6)^.

Women living with HIV suffer from the stigma associated with the virus, as a positive diagnosis affects their leisure, sexual experiences, work, and relationships, in addition to compromising their physical and mental health. The vulnerability of women to HIV is tripled when combined with economic and racial issues^(7)^.

In 1996, the approval of Law No. 9,313 regulated the free distribution of combined antiretrovirals for PLHIV through SUS. It is worth noting that Brazil was the first developing country to adopt this measure, providing individuals diagnosed with HIV the opportunity for timely treatment^(8)^. Adherence to ART, as well as undergoing the necessary tests for diagnosis, is crucial for treatment success and quality of life. Late diagnosis and interruptions in antiretroviral (ARV) treatment can lead to a decrease in CD4+ T-cell counts, an increase in viral load, opportunistic infections, and progression to AIDS, which can result in death^(9,10)^.

The HIV infection impacts both society and the economy, in both the formal and informal sectors. Stigma and fear of discrimination are frequently experienced by PLHIV, especially women, particularly when the immune system is compromised, leading to the development of opportunistic infections and the perception of physical death and social exclusion^(6,11)^.

In this light, combined with the socio-affective and sexual discrimination constantly faced by PLHIV, there is a notable increase in the risk of psychological suffering and the manifestation of mental disorders. This can lead to reduced adherence to antiretroviral treatment and further exacerbate the mental suffering caused by the diagnosis and the search for treatment^(12)^.

Given the importance of the topic, the study poses the following guiding question: What is the perception of women living with HIV regarding their diagnosis, antiretroviral treatment, and mental health?

## OBJECTIVES

To understand the perception of women living with HIV regarding their diagnosis, treatment, and mental health.

## METHODS

### Ethical aspects

The study was approved by the Research Ethics Committee of the Federal University of Alagoas and followed the guidelines and regulations of the Brazilian Ministry of Health Resolutions No. 466/12 and 510/16. All participants agreed to take part in the study by signing the Informed Consent Form (ICF). To maintain the anonymity and privacy of the participants, their names were replaced by alphanumeric codes, consisting of the letter “P” (for “patient”) followed by a cardinal number.

### Type of study

This is a descriptive study with a qualitative approach, utilizing Jean Watson’s Theory of Human Caring as the theoretical framework.

### Theoretical framework

Jean Watson’s Theory of Human Caring provides a philosophical and scientific foundation for nursing practice, emphasizing care and empathy. The theory posits that human care is culturally essential for survival and moves beyond a strictly biomedical approach by recommending the application of the ten elements of the Caritas Process:

1. Practice love, temperance, and impartiality in self-care;
2. Be present in your practice and reinforce deep beliefs;
3. Cultivate spiritual and “transpersonal self” practices, transcending the ego;
4. Develop and maintain a trusting, authentic helping relationship;
5. Support the expression of both positive and negative feelings, connecting deeply with your spirit and that of the person being cared for;
6. Be creative, using various forms of knowledge in the care process, engaging in artistic care and restoration practices;
7. Engage in a genuine teaching-learning experience, considering the unity of the being and its meanings, staying aligned with the other’s perspective;
8. Create a healing environment where the individual’s wholeness, beauty, comfort, dignity, and peace are enhanced;
9. Assist with basic needs, with intentional consciousness of care, offering what is essential to human caring;
10. Maintain openness and attention to spiritual phenomena and the existential dimensions of life and death, caring for both your soul and the soul of the person being cared for^(13)^.

In this context, the theory was used to guide professionals in viewing, after gaining knowledge of the perceptions of women living with HIV, the human being in its complete essence, moving beyond the focus on curative technology and highlighting the importance of care. In this way, it is understood that care belongs to social phenomenology but can only be effective when practiced interpersonally, as human beings are emotional creatures with personal conflicts, crises, illnesses, needs, and natural interactions^(13)^.

### Methodological Procedures

A semi-structured instrument was used in the study, containing themes related to the research topic. To ensure greater reliability and test the instrument’s applicability, two preliminary interviews were conducted with the target audience. After evaluating the instrument and verifying its applicability, the research was conducted. Invitations to participate were made through individual contact with the women in the waiting room for consultations. During this contact, a brief explanation of the research objectives and procedures was provided.

Once consent was obtained, the women were offered a designated room provided by the facility for data collection, a private space that ensured confidentiality during the interview, in case the participant felt uncomfortable answering questions in the waiting room. At that time, the informed consent form (ICF) was read and signed, and subsequently, the interview was conducted using the semi-structured instrument.

### Study Setting

The study was conducted at a SAE for PLHIV/AIDS in a capital city in Northeast Brazil. This site was chosen because it serves PLHIV/AIDS from across the state and operates year-round.

### Data Source

The study included women aged 18 and older who were undergoing ART and were registered at the SAE. Women who presented physical, emotional, and/or behavioral vulnerabilities were excluded from the study after a brief assessment by the data collection team.

Participant selection was done by convenience sampling, and data collection was concluded when data saturation was achieved—that is, when repetition of information occurred in the interviews, meeting the research objective. Identifying saturation is necessary to conclude data collection and determine the appropriate number of participants for qualitative research. Saturation indicates when the empirical material begins to show redundancy and repetition from the interviewer’s perspective. In this context, eight women participated in the study^(14)^.

### Data Collection and Organization

Data collection took place between November and December 2022. For gathering information, semi-structured individual interviews were conducted using a recorder and a script organized into two sections:

a) Characterization of the women, including sociodemographic data such as age, race/ethnicity, marital status, number of children, sexual orientation, education level, occupation, and household income. Regarding clinical data, information was collected on the year of diagnosis, mode of transmission, knowledge of viral load, diagnosis of psychiatric/mental illness, and the use of psychiatric medication;
b) Questions related to diagnosis, treatment, and mental health, including: “How was the moment when you received your diagnosis, and how did you accept the new routine of continuous medication use?”; “Do you believe there is anything that makes adherence to antiretroviral therapy more difficult?”; “In your opinion, how could healthcare professionals help prevent the discontinuation of medication?”.

To ensure the safety and quality of data collection, the study followed the guidelines outlined in the *Consolidated Criteria for Reporting Qualitative Research* (COREQ)^(15)^.

### Data Analysis

The interviews were fully transcribed to facilitate reflection on the topics discussed. Data analysis was conducted using Content Analysis as proposed by Laurence Bardin^(16)^. In this approach, understanding the discourse is derived from language, with quantifiable methods used to report the content of the messages. Thus, the spoken discourse in the interviews or observed by the researcher is classified into categories that aid in understanding the narratives.

Furthermore, the content analysis process consists of three phases:

1. Pre-analysis, which involves floating reading, document selection, reformulation of objectives and hypotheses, and formulation of indicators;
2. Material exploration, aimed at defining categories, grouping, and coding common characteristics;
3. Data processing, involving the interpretation of codes and drawing inferences.

Bardin’s technique reveals the relationships between the content and the outside world through a “deep” reading. This process uses linguistic systems to access ideas and knowledge, allowing practitioners to understand and apply the analyzed content.

After organizing the content, the characterization of the women and the identification of three categories were carried out, reflecting the women’s perceptions regarding diagnosis, treatment, and mental health:

1. Feelings about the discovery of the disease and the acceptance of treatment;
2. Impact on the mental health of women living with HIV and their relationship with treatment;
3. The role of nursing professionals in consultations with PLHIV, discussed in light of Jean Watson’s Theory of Human Caring.

## RESULTS

The presentation of the results of this study is divided into two sections: the first focuses on the characterization of the participants, and the second addresses the thematic categories.

### Characterization of the participants

The eight women in this study ranged in age from 18 to 65, with most being 50 years old or older. All identified as heterosexual, with a predominance of participants identifying as mixed race (*parda*), and the majority were divorced. Most had completed high school. They were not engaged in paid work, with a monthly per capita household income of up to one minimum wage, derived from government assistance programs or self-employment. They had between one and four children. The most commonly reported mode of transmission was sexual contact, with most diagnoses occurring between 2016 and 2021.

Most of the women were receiving mental health support and/or using psychotropic medication.

### Feelings about the discovery of the disease and its relationship with treatment

From the testimonies provided, feelings of uncertainty and despair were evident following the discovery of a disease burdened with stigma. These feelings were compounded by the moral judgment experienced, including from healthcare professionals, abandonment by their partners, and the emergence of self-destructive thoughts.

*I just cried, I was in despair. I didn’t expect it, I couldn’t accept it. I didn’t have support; the nurse didn’t know how to assist me or explain, I was left without direction, and my husband abandoned me* [...]. (P2)

*So, for me, it was like the ground had been pulled out from under me, like there was no other way to live, and that was it* [...]. (P5)

*I lost it; my mind was in chaos. The medication, the situation, and a lot of therapy to accept it all* [...]. (P7)

### Impact on the mental health of women living with HIV/AIDS and their relationship with healthcare professionals

In this category, the women’s responses highlight the impact on their mental health, often described as symptoms of anxiety that affect their daily lives and medication adherence, sometimes leading to partial or complete discontinuation.

[...] *The fear of dying, right* [...] *the fear of tomorrow, in this case. I think it’s more about the anxiety of wanting to know what might happen tomorrow. Unfortunately, anxiety causes this fear of the future, right* [...] *the fear that things won’t go right, wondering what the future will be like* [...] *wanting to know the future, even though we don’t know* [...]. (P5)

[...] *Sometimes you just want to give up. I stopped taking it myself for an entire month, I didn’t want it anymore* [...]. *I went back to taking it because I felt the effects of not having the medication, and I felt guilty, so I wanted to take it again.* (P1)

[...] *I thought about killing myself when I got the diagnosis, and even today I don’t accept the medication or the result very well.* (P8)

[...] *I came to understand the reason for contracting HIV. I think the purpose was to seek psychological help because, without a diagnosis of a disease, it’s not easy to get psychological help like that.* (P5)

### The role of healthcare professionals in caring for PLHIV/AIDS

The role of healthcare professionals in caring for PLHIV/AIDS is to establish and create an individualized experience through the professional-patient relationship throughout the course of the illness, helping patients overcome the interference of physical, psychological, cultural, spiritual, socioeconomic, and disease-related factors, as well as those related to treatment.

[...] *I think human warmth is essential. Holding someone’s hand and saying ‘it’s going to be okay,’ telling them that this is a new chapter they will live through differently, and that they will learn to adapt to it.* (P2)

[...] *Not judging. I would encourage treatment, including psychiatric treatment, like in my case now, to help improve things, encourage them.* (P6)

[...] *Help me by giving me strength, talking to me, with advice, a visit, and helping not just me, but my grandchildren as well.* (P3)

[...] *For example, explaining the importance of the medication, because without it, they won’t have any resistance to anything, and that’s important. I talk, I explain, I give examples. As a citizen, the nurse has to think of themselves as if they were that citizen too.* (P7)

## DISCUSSION

### Characterization of the Research Participants

Regarding race/ethnicity, among the eight women interviewed, six self-identified as mixed race (*parda*). Although these numbers align with national statistics, white women represent the second largest group. This data is important for the study, as race/ethnicity directly influences healthcare relationships due to the risk of institutional racism faced by Black and mixed-race individuals, which can increase the chances of morbidity and mortality from HIV/AIDS^(17,18)^.

The marital status of the participants is relevant to the study because it contributes to understanding their response to coping with the disease, indicating whether there is social and economic support, as well as enabling the tracking of transmission modes and treatment for possible partners. Being divorced or separated may suggest a condition of social vulnerability. The condition of being single may be related either to self-imposed social isolation or rejection by potential partners^(19)^.

Regarding the women who identified as heterosexual, a study showed that the majority of the patients surveyed identify as heterosexual. This number is even more significant among the female population, confirming that heterosexual transmission is the most common mode among women, highlighting the need for investment and strengthening specific campaigns for this population^(20)^.

With regard to motherhood, this concept is relevant to the study because the desire of women to protect their children from potential discrimination, due to the negative stereotypes associated with PLHIV, can generate a sense of resilience and hope. This sentiment helps them overcome the anxiety caused by the discovery of the disease, their experience with it, and the concern about death. Motherhood can act as a positive factor, encouraging women to continue ART due to the desire to see their children grow and be part of their upbringing^(12)^.

Education is an important factor in analyzing treatment adherence and effectiveness, as there is a direct relationship between education level and the ability to access and understand information related to HIV/AIDS. Additionally, a higher level of education can increase the chances of better employment opportunities, both financially and mentally. On the other hand, a low level of education is associated with difficulty in understanding what the virus is, the importance of ART, and its implications for life^(20,21)^.

Regarding employment, the majority of the interviewees reported being unemployed. For the purposes of this study, occupation directly influences financial aspects. Historically, women have been part of the population engaged in domestic work, either in their own homes or caring for other families. Although this scenario is changing, many such cases still exist in Brazil. Women dedicated exclusively to household work may suffer from financial dependence on their husbands or families, which increases their vulnerability to violence, as well as impacting their mental and physical health. The stigma associated with HIV can lead to exclusion from the labor market, causing negative effects on individual and/or family income^(17)^.

Regarding data related to the condition, information was analyzed on the year of diagnosis, mode of transmission, and whether the participants were receiving support from a psychologist or psychiatrist, with or without medication.

In this context, it was observed that the majority of participants were diagnosed between 2016 and 2021, with sexual transmission being the most frequent mode. These data are consistent with the epidemiological bulletin, which, when analyzing the years from 2007 to 2022, showed that the period between 2016 and 2021 had the highest number of HIV cases reported in the Notifiable Diseases Information System (SINAN in Portuguese) for both female and male populations. The mode of transmission is an important factor in this study, as it allows for an understanding of possible failures in the promotion and adherence to barrier prevention methods, especially among the portion of the population that believes they do not need to use such methods, such as married women who consider themselves outside the “at-risk group”^(12)^.

Regarding psychological or psychiatric support, the majority of the women confirmed receiving this type of care. Mental health is of utmost importance in the daily lives of women living with HIV, as living with an incurable disease that requires continuous medication, in addition to the uncertainties related to daily vulnerabilities, can trigger symptoms like anxiety and depression. Psychological support helps recognize feelings such as self-hatred or resentment toward the partner who caused the exposure, guilt for not having taken preventive measures, a negative perception of the disease, fear of death, and thoughts about the impossibility of a cure—factors that can affect mental health and lead to the appearance of depressive and anxious symptoms^(21)^.

When asked about the use of psychotropic medications, seven of the eight interviewees confirmed daily use of such medications. Patients with HIV and mental health issues require greater attention and care when adhering to ART, as the use of psychiatric medication is negatively associated with adherence to ART due to stigma and the influence of additional drugs on the body. This is because the introduction of another medication regimen, in addition to antiretrovirals, highlights the need for an additional daily medication routine, requiring an analysis of possible drug interactions, as well as the side effects that psychiatric medications can cause. These factors can influence the individual’s self-perception and their adherence to ART^(21,22)^.

### Feelings about the discovery of the disease and its relationship with treatment

This study found that women are vulnerable in various ways, and exposure to HIV is no exception. In addition to the neglect faced by this population, underreporting of cases also contributes to the increased incidence of HIV within this demographic^(10,12)^.

With the emergence of the HIV epidemic in the 20th century, a “risk group” was defined for the acquisition of the virus, which led to prejudice against this population. According to popular belief, the disease only affected people who were homosexual, engaged in promiscuous behavior, or belonged to the upper-middle class^(20)^.

As a result, PLHIV began to experience self-blame, undergoing various processes of acceptance following their diagnosis. The difficulties and delays in medicine recognizing women as vulnerable to infection allowed HIV/AIDS to grow silently, reinforcing gender inequalities. Based on the participants’ statements, feelings of uncertainty and despair stand out after discovering a disease laden with stigma, combined with the suffering caused by moral judgment, including from healthcare professionals, abandonment by partners, and even the emergence of suicidal thoughts^(19)^.

The diagnosis of HIV infection is considered a profoundly negative event in people’s lives, as it is associated with a stigmatized disease in society, affecting family and social dynamics, and often causing isolation and abandonment from oneself and others^(5)^.

Margaret Jean Watson’s Transpersonal Theory of Human Caring highlights, in the women’s statements, the need to provide holistic care, recognizing that humans are emotional beings with personal and external crises, and varying levels of difficulty in accepting the disease^(12,15)^.

It is evident that adherence to treatment is essential for controlling the virus in the body, preventing progression to the AIDS phase, and reducing the chances of opportunistic infections. Treatment adherence is crucial for the viral load to become undetectable, with fewer than 40 copies/mL of the virus in the blood. For this to happen, the therapeutic regimen must achieve an adherence rate of 95% or higher of prescribed doses^(1)^.

### Impact on the mental health of Women Living with HIV/AIDS and their relationship with healthcare professionals

PLHIV, without proper support, can develop psychiatric conditions or experience a worsening of pre-existing conditions such as depression and pathological anxiety. This can hinder treatment adherence in various ways, making it more difficult to improve quality of life and increase survival rates^(21)^.

Based on the participants’ responses, it is evident that symptoms such as anxiety and depression affect patients’ daily lives, impairing medication adherence and, in some cases, leading to partial or complete discontinuation of treatment. Additionally, the fear of death is an extreme form of anxiety that profoundly impacts people’s lives and decisions. For this reason, these symptoms must be recognized in time so that appropriate care can be initiated, allowing PLHIV to receive the necessary support for their treatment, without abandoning or delaying it, which would further exacerbate the disease^(8)^.

When healthcare professionals overlook these symptoms, it can worsen the clinical condition, as individuals living with HIV/AIDS may feel neglected in terms of their biopsychosocial aspects^(22)^. Minimizing these feelings contributes to isolation, low self-esteem, loneliness, anxiety, depression, and diminished hope, which may lead to treatment abandonment. Thus, it is clear that good quality of life can make a significant difference in the patient’s acceptance of and personal conduct regarding treatment^(8,21)^.

In any therapy, the initial phase of treatment is the most challenging for adherence due to the adaptation to a new daily medication routine and the side effects. PLHIV develop resilience mechanisms to consistently continue treatment^(11)^.

Healthcare professionals must reinforce and support the patient’s belief system as a mechanism for emotional control, facilitating the development of trust and understanding between the patient and professional. From the perspective of the Transpersonal Theory of Human Caring, care practices should promote the expression and acceptance of both positive and negative feelings, encouraging a system of humanistic and altruistic values^(21)^.

In the long term, the patient’s difficulty in coping with their disease results in harm to their own treatment. This impairs the patient’s ability to handle additional stress caused by the HIV diagnosis. Therefore, the main idea behind the concept of adherence is how the person living with HIV/AIDS responds to the healthcare professional’s recommendations and how they adapt to the routine of a given therapeutic regimen in their daily life^(1,11)^.

Analyzing the participants’ statements, there is a clear need for effective communication, active listening, and a non-judgmental approach. In this way, the healthcare professional should foster a relationship that builds deep trust and understanding, facilitating medication adherence by addressing the reasons for possible refusal and taking decisive action. By analyzing the situation and the difficulties expressed by the patient through dialogue, the healthcare professional should observe the patient’s needs, listen to their concerns, identify problems, and plan actions together to promote overall well-being, considering the patient’s lived reality^(21,22)^.

### Study limitations

The limitations of the study are related to the COVID-19 pandemic, which affected the data collection period by reducing the number of available professionals and patients being treated. To complete data collection, the researcher adopted strategies such as the use of personal protective equipment (PPE) and scheduling interviews on days and times coordinated with the women.

### Contributions to the Field of Nursing

The study significantly contributes to the care of the mental health of women living with HIV/AIDS, emphasizing the importance of empathetic assistance for adherence to antiretroviral therapy. The approach should ensure that all patients can benefit from the treatment without being subjected to the stigma associated with PLHIV. Additionally, the study proposes a humanized care model that promotes strategies to address vulnerabilities and improve the quality of care provided.

## FINAL CONSIDERATIONS

Based on the results of this study, it is concluded that women living with HIV face a moment of great fragility, becoming even more vulnerable during the process of discovering the infection, especially due to the impact the virus has on their individual and collective dynamics. The anxiety surrounding death and the fear of prejudice from family, society, and even healthcare professionals generate feelings of despair, abandonment, and, in some cases, denial of the disease.

Moreover, the study reveals that mental health has a considerable influence on adherence to antiretroviral medication. The discovery of a chronic, incurable, and stigmatized disease in society can trigger psychological issues or exacerbate pre-existing conditions such as anxiety and depression. Thus, pathological anxiety and depression are symptoms that affect patients’ daily actions, influencing adherence to their medication routine. This can negatively impact viral load, leading to the emergence of opportunistic diseases, progression to AIDS, and even death.
