# Supplementary material for: Women living with HIV: perception regarding diagnosis, treatment and mental health
Source: Rev Bras Enferm. 2025 Mar 17;78(Suppl 2):e20240134. doi: 10.1590/0034-7167-2024-0134 (PMC11913424; doi:10.1590/0034-7167-2024-0134)
Supplement: Translation

# Translation: Mulheres vivendo com HIV: percepção acerca do diagnóstico, tratamento e da saúde mental

**DOI:** 10.1590/0034-7167-2024-0134pt

**Authors:** Aline de Lima Barbosa, Amuzza Aylla Pereira dos Santos, Christefany Régia Braz Costa, Tâmara Silva de Lucena, Viviane dos Santos Melo, Lea Christine Ferreira de Araújo, Davi Porfirio da Silva, Dayse Carla Alves Sales Pereira

**Affiliations:** I Universidade Federal de Alagoas. Maceió, Alagoas, Brasil

**Keywords:** Percepção, Mulheres, HIV, Adesão ao Tratamento, Antirretroviral

## Abstract

**Objetivos:**

conhecer a percepção das mulheres vivendo com HIV acerca do diagnóstico, tratamento e da saúde mental.

**Métodos:**

trata-se de um estudo qualitativo, realizado com oito mulheres, no período de novembro a dezembro de 2022. Para a análise dos dados, utilizou-se a técnica de Análise de Conteúdo de Bardin, a partir dos discursos das participantes, em conjunto com a Teoria do Cuidado Humano de Jean Watson.

**Resultados:**

o conteúdo foi organizado em categorias, a saber: Sentimentos diante da descoberta da doença e sua relação com o tratamento; Impacto na saúde mental de mulheres vivendo com HIV/Aids e sua relação com os profissionais de saúde.

**Considerações Finais:**

conclui-se, por meio dos discursos, que a percepção das mulheres com HIV em relação ao diagnóstico, tratamento e à saúde mental é marcada por um momento de fragilidade, tornando-as vulneráveis no processo de descoberta da infecção, principalmente por afetar toda a sua dinâmica individual e coletiva.

## INTRODUÇÃO

A Síndrome da Imunodeficiência Adquirida (SIDA; aids – *Acquired Immunodeficiency Syndrome*) foi identificada na década de 1970, nos Estados Unidos da América (EUA). Trata-se de uma condição crônica associada à infecção pelo Vírus da Imunodeficiência Humana (HIV), e constitui um agravo à saúde pública mundial, comprometendo o sistema imunológico, responsável pela defesa do organismo contra doenças^(1,2)^.

Com o avanço da ciência e a introdução da terapia antirretroviral (TARV), a realidade das PVHIV foi modificada, havendo uma redução da morbimortalidade. Isso proporcionou aos infectados pelo vírus não apenas maior expectativa de vida, com o aumento da sobrevida, mas também qualidade de vida, permitindo a reconstrução de seus projetos pessoais, incluindo relacionamentos amorosos e a construção de uma família^(3)^.

Nesse contexto, o diagnóstico precoce desse agravo visa contribuir para a redução da morbimortalidade, mas, principalmente, para apoiar a reconstrução dos projetos pessoais das PVHIV, que ainda não identificaram a doença. O diagnóstico precoce possibilita o tratamento oportuno e o seguimento adequado^(4)^. Dessa forma, visando proporcionar maiores oportunidades de cuidado às PVHIV, o Sistema Único de Saúde (SUS) instituiu, na década de 1980, os Serviços de Atenção Especializada (SAE)^(5,6)^.

As mulheres que vivem com HIV sofrem com a estigmatização do vírus, pois o diagnóstico positivo interfere no lazer, na vivência da sexualidade, no trabalho e em seus relacionamentos, além de comprometer sua saúde física e psíquica. A vulnerabilidade ao HIV entre as mulheres é triplicada quando somada às questões econômicas e raciais^(7)^.

Em 1996, ocorreu a aprovação da Lei n. 9.313, que regulamenta a distribuição gratuita de antirretrovirais combinados para PVHIV no SUS. Vale salientar que o Brasil foi o primeiro país em desenvolvimento a adotar essa medida, proporcionando às pessoas diagnosticadas com HIV a oportunidade de tratamento oportuno^(8)^. A adesão à TARV, bem como a realização dos testes que permitem o diagnóstico, são de suma importância para o sucesso do tratamento e a qualidade de vida. O diagnóstico tardio e a interrupção dos antirretrovirais (ARV) podem implicar na diminuição da contagem dos linfócitos TCD4+, no aumento da carga viral, no adoecimento por infecções oportunistas e na evolução para AIDS, podendo levar à morte^(9,10)^.

A infecção pelo HIV impacta a sociedade e a economia, tanto no setor formal quanto no informal. O estigma e o medo da discriminação são frequentemente vivenciados pelas PVHIV, especialmente pelas mulheres, principalmente quando há o agravamento do sistema imunológico, favorecendo o desenvolvimento de infecções oportunistas e levando à percepção de morte física e exclusão social^(6,11)^.

Sob essa perspectiva, associada ao preconceito e à discriminação socioafetiva e sexual constantemente experimentados pelas PVHIV, é notável o aumento do risco de sofrimento psicológico e da manifestação de transtornos mentais. Isso pode ocasionar a redução da adesão aos antirretrovirais, além de agravar o sofrimento mental causado pelo diagnóstico e pela busca por tratamento^(12)^.

Diante da relevância do tema, o estudo traz a seguinte pergunta norteadora: qual é a percepção das mulheres vivendo com HIV acerca do diagnóstico, do tratamento com antirretrovirais e de sua saúde mental?

## OBJETIVOS

Conhecer a percepção das mulheres vivendo com HIV acerca do diagnóstico, do tratamento e da saúde mental.

## MÉTODOS

### Aspectos éticos

O estudo foi aprovado pelo Comitê de Ética em Pesquisa (CEP) da Universidade Federal de Alagoas e seguiu as diretrizes e normas das Resoluções do Ministério da Saúde nº 466/12 e 510/16. Todas as mulheres aceitaram participar da pesquisa mediante a assinatura do Termo de Consentimento Livre e Esclarecido (TCLE). Para manter o anonimato e a privacidade das participantes da pesquisa, seus nomes foram substituídos por códigos alfanuméricos, formados pela letra “P” (de “paciente”), seguida de um numeral cardinal.

### Tipo de estudo

Trata-se de um estudo descritivo, com abordagem qualitativa, no qual foi utilizada como referencial teórico a Teoria do Cuidado Humano de Jean Watson.

### Referencial teórico

A Teoria do Cuidado Humano de Jean Watson estabelece como referencial para a atuação da enfermagem a filosofia e a ciência direcionadas ao cuidado e à empatia, pois define que o cuidado humano é culturalmente necessário para a sobrevivência. Essa teoria rompe com o conceito estritamente biomédico, recomendando a aplicação dos dez elementos do Processo Caritas:

1. Praticar o amor, a temperança e a imparcialidade no cuidado de si;
2. Estar presente em sua atuação e revigorar crenças profundas;
3. Cultivar práticas espirituais e do “eu transpessoal”, ultrapassando o ego;
4. Desenvolver e manter uma relação de ajuda-confiança no cuidado autêntico;
5. Apoiar a expressão de sentimentos positivos e negativos, conectando-se profundamente com o próprio espírito e com o da pessoa cuidada;
6. Ser criativo, utilizando diversas formas de conhecimento no processo de cuidar, engajando-se em práticas artísticas de cuidado e reconstituição;
7. Engajar-se em uma experiência genuína de ensino-aprendizagem, considerando a unidade do ser e seus significados, mantendo-se alinhado ao referencial do outro;
8. Criar um ambiente de reconstituição (*healing*), no qual a totalidade do indivíduo, a beleza, o conforto, a dignidade e a paz sejam potencializados;
9. Auxiliar nas necessidades básicas, com consciência intencional de cuidado, oferecendo o que é essencial ao cuidado humano;
10. Manter abertura e atenção aos fenômenos espirituais e dimensões existenciais da vida e morte, cuidando tanto da própria alma quanto da alma da pessoa cuidada^(13)^.

Nesse contexto, a teoria foi utilizada para guiar o profissional a enxergar, após o conhecimento da percepção das mulheres vivendo com HIV, o ser humano em sua essência completa, indo além da valorização da tecnologia curativa e destacando a importância do cuidado. Dessa forma, entende-se que o cuidado pertence à fenomenologia social, mas só será efetivo se praticado de forma interpessoal, visto que os humanos são seres emocionais, com conflitos pessoais, crises, doenças, necessidades e interações por natureza^(13)^.

### Procedimentos Metodológicos

Utilizou-se, para o estudo, um instrumento semiestruturado contendo os temas relacionados ao objeto de estudo. A fim de obter maior confiabilidade e testar a aplicabilidade do instrumento, foram realizadas duas entrevistas prévias com o público-alvo. Após a avaliação do instrumento e a verificação de sua aplicabilidade, a pesquisa foi desenvolvida. O convite para a participação foi feito por meio de contato individual com as mulheres na sala de espera para as consultas. Nesse contato, também foi realizada uma breve explicação sobre o objetivo da pesquisa e sua execução.

Após o aceite, foi oferecida às mulheres uma sala específica, disponibilizada pela unidade para a coleta de dados, um local reservado que proporcionava privacidade para a realização da entrevista, caso a participante não se sentisse confortável para responder às perguntas na sala de espera. Nesse momento, foi realizada a leitura e assinatura do TCLE e, posteriormente, a entrevista foi conduzida com o uso do instrumento.

### Cenário do Estudo

O estudo foi realizado em um SAE para PVHIV/aids, em uma capital do Nordeste do Brasil. O cenário foi escolhido por acolher PVHIV/aids de todo o estado e funcionar durante o ano inteiro.

### Fonte de Dados

Foram incluídas no estudo mulheres com idade igual ou superior a 18 anos, que estavam submetidas à TARV e cadastradas no SAE. Foram excluídas as mulheres que apresentaram fragilidades físicas, emocionais e/ou comportamentais, após uma breve avaliação junto à equipe do local de coleta de dados.

A seleção das participantes do estudo ocorreu por conveniência, e a finalização da coleta de dados foi feita mediante a saturação das informações, ou seja, quando houve repetição dos dados nos discursos, atendendo ao objetivo proposto da investigação. A identificação da saturação é necessária para encerrar a coleta de informações e determinar o número adequado de participantes para pesquisas qualitativas. A saturação põe fim à coleta de dados, pois demonstra quando o material empírico começa a apresentar redundância e repetição, sob o ponto de vista da entrevistadora. Nesse contexto, participaram do estudo oito mulheres^(14)^.

### Coleta e organização dos dados

A coleta de dados ocorreu entre os meses de novembro e dezembro de 2022. Para a produção das informações, utilizou-se a técnica da entrevista semiestruturada de forma individual, com o uso de gravador e um roteiro organizado em duas seções:

a) Caracterização das mulheres, com dados sociodemográficos como idade, raça/cor, estado civil, filhos, orientação sexual, escolaridade, profissão e renda familiar. No que se refere aos dados clínicos, coletaram-se informações sobre o ano do diagnóstico, forma de transmissão, conhecimento sobre a carga viral, diagnóstico de doença psiquiátrica/mental e uso de medicação psiquiátrica;
b) Questões referentes ao diagnóstico, ao tratamento e à saúde mental: “Como foi o momento do resultado do diagnóstico para você e a aceitação da nova rotina com uso da medicação contínua?”; “Você acredita que há algo que dificulte a adesão à terapia antirretroviral?”; “Na sua visão, como o profissional de saúde poderia contribuir para evitar a desistência do uso da medicação?”.

Para garantir a segurança e a qualidade da coleta de dados, o estudo seguiu os critérios presentes no *Consolidated Criteria for Reporting Qualitative Research* (COREQ)^(15)^.

### Análise dos dados

As entrevistas foram transcritas na íntegra para facilitar a reflexão sobre os temas abordados. A análise dos dados foi realizada por meio da Análise de Conteúdo, conforme proposta por Laurence Bardin^(16)^. Nessa abordagem, a compreensão do discurso é derivada da linguagem, utilizando-se métodos quantificáveis para relatar o conteúdo das mensagens. Assim, o discurso falado nas entrevistas ou observado pelo pesquisador é classificado em categorias que auxiliam na compreensão dos relatos.

Além disso, o processo de análise de conteúdo é composto por três fases:

1. Pré-análise, que envolve: leitura flutuante; seleção dos documentos; reformulação de objetivos e hipóteses e formulação de indicadores;
2. Exploração do material, com o objetivo de definir categorias, agrupamento e codificação de características comuns;
3. Tratamento dos dados, com a interpretação dos códigos e inferências.

A técnica de Bardin revela as relações entre o conteúdo e o mundo exterior por meio de uma leitura “profunda”. Esse processo utiliza sistemas linguísticos para acessar ideias e conhecimentos, permitindo que os praticantes compreendam e utilizem o conteúdo analisado.

Após a organização do conteúdo, foram realizadas a caracterização das mulheres e a identificação de três categorias que refletiram a percepção das mulheres sobre o diagnóstico, o tratamento e a saúde mental:

1. Sentimentos em relação à descoberta da doença e à aceitação do tratamento;
2. Impacto na saúde mental de mulheres vivendo com HIV e sua relação com o tratamento;
3. A atuação do profissional de enfermagem na consulta das PVHIV, discutida à luz da Teoria do Cuidado Humano de Jean Watson.

## RESULTADOS

A apresentação dos resultados deste estudo foi dividida em dois momentos: o primeiro refere-se à caracterização das participantes da pesquisa e o segundo às categorias temáticas.

### Caracterização das participantes da pesquisa

As oito mulheres participantes deste estudo tinham idades entre 18 e 65 anos, sendo que a maior parte possuía 50 anos ou mais. Todas se identificaram como heterossexuais, com predominância da cor parda, estado civil divorciadas, e a maioria tinha o ensino médio completo. Elas não exerciam atividades laborais remuneradas, possuindo, assim, renda familiar mensal per capita de até um salário mínimo, proveniente de programas governamentais ou de serviços prestados como autônomas. Tinham entre um e quatro filhos. A forma de transmissão relatada pela maioria foi a via sexual, e o diagnóstico da maioria ocorreu entre os anos de 2016 e 2021.

A maior parte das mulheres realizava algum acompanhamento relacionado à saúde mental e/ou fazia uso de medicação psicotrópica.

### Os sentimentos frente à descoberta da doença e a relação com o tratamento

A partir dos depoimentos presentes no estudo, evidenciam-se as incertezas e o sentimento de desespero diante da descoberta de uma doença que sofre tanto preconceito. Esse sentimento está aliado ao sofrimento causado pelo julgamento moral, inclusive por parte de profissionais, ao abandono por parte do companheiro e até mesmo ao surgimento de pensamentos autodestrutivos.

*Só chorava, entrei em desespero. Não esperava, não aceitava isso. Não tive suporte, a enfermeira não soube me atender e explicar, fiquei sem chão e fui abandonada pelo meu marido* [...]. (P2)

*Então, pra mim foi como se tivesse tirado o meu chão, como se não tivesse mais uma forma de viver e fosse só aquilo* [...]. (P5)

*Pirei, minha cabeça ficou bagunçada. A medicação, a situação e muita terapia para aceitar* [...]. (P7)

### Impacto na saúde mental de mulheres vivendo com HIV/aids e sua relação com o profissional de saúde

Nesta categoria, evidencia-se, pelas respostas das mulheres, o impacto na sua saúde mental, que pode ser descrito como sintomas de ansiedade que afetam o cotidiano das pacientes e a adesão à medicação, levando inclusive a situações de desistência parcial ou total.

[...] *o medo de morrer né* [...] *o medo do amanhã, no caso. Eu acho que diz mais a respeito da ansiedade de querer saber o que pode acontecer amanhã, infelizmente a ansiedade causa esse medo do amanhã né* [...] *o medo das coisas não dar certo de como vai ser o futuro* [...] *de querer saber o futuro sendo que a gente não* [...]. (P5)

[...] *Tem hora que você quer desistir, eu mesmo desisti por um mês inteiro, não queria mais* [...]. *Voltei a tomar por sentir efeitos pela falta do remédio, senti vontade de tomar porque pesou na consciência*. (P1)

[...] *Pensava em me matar quando recebi isso e inté hoje eu não aceito bem remédio e o resultado.* (P8)

[...] *eu vim entender o porque do propósito de eu ter pego HIV, de ter contraído HIV, acho que mais o propósito foi buscar ajuda psicológica, porque. quem não tem um diagnóstico de uma doença, não tem uma ajuda psicológica assim tão fácil.* (P5)

A atuação do profissional de saúde no cuidado com a PVHIV/aids visa estabelecer e criar uma experiência individual através da vivência da relação profissional-usuário durante toda a trajetória da enfermidade, ajudando-o a superar as interferências de fatores físicos, psicológicos, culturais, espirituais, socioeconômicos, das condições da doença e do tratamento.

[...] *Eu acho que o calor humano é essencial. Segurar na mão e dizer “vai dar certo”, informar que agora é uma nova história que vai viver diferente e que vai aprender a se adaptar a isso.* (P2)

[...] Não julgar. Eu iria incentivar o tratamento, o psiquiátrico também, como é o meu caso agora, para as coisas melhorar, incentivar. (P6)

[...] *Me ajudar dando uma força, conversa, em tudo, um conselho, uma visita em tudo e ajudando não só eu, os meus netos também*. (P3)

[...] *Um exemplo: explicar que a importância do medicamento, fora que ele sem o medicamento, ele não vai ter resistência pra nada, isso é importante. Eu converso, falo, dou exemplo, como cidadã, o enfermeiro tem que pensar como se fosse esse cidadão também.* (P7)

## DISCUSSÃO

### Caracterização das participantes da pesquisa

No que diz respeito à raça/cor, entre as oito mulheres entrevistadas, seis se autodeclararam pardas. Embora esses dados estejam alinhados com as estatísticas nacionais, a população feminina autodeclarada branca aparece como o segundo maior grupo. Esse dado é importante para o estudo, visto que a raça/cor influencia diretamente a relação de atendimento à população, em função do risco de racismo institucional enfrentado pelas pessoas pretas e pardas, o que pode elevar as chances de morbimortalidade por HIV/aids^(17,18)^.

O estado civil das participantes é relevante para o estudo, pois contribui para a compreensão da resposta ao enfrentamento da doença, indicando se há ou não suporte social e econômico, além de permitir o rastreamento da forma de transmissão e o tratamento de possíveis parceiros. O status de divorciada ou separada pode sugerir uma condição de vulnerabilidade social. A condição de estar solteira pode estar relacionada tanto ao autoimposto isolamento social quanto à rejeição por possíveis parceiros^(19)^.

Com relação às mulheres que se declararam heterossexuais, um estudo mostrou que a maioria dos pacientes pesquisados se identifica como heterossexual. Esse número é ainda mais expressivo entre a população feminina, confirmando que a transmissão heterossexual é a mais comum entre as mulheres, o que populariza a epidemia nesse grupo e destaca a necessidade de investimentos e o fortalecimento de campanhas específicas para essa população^(20)^.

No que tange à maternidade, esse conceito é relevante para o estudo, pois o desejo das mulheres de protegerem seus filhos diante da possibilidade de discriminação, devido aos estereótipos negativos prevalentes sobre portadores de HIV/aids, pode gerar um sentimento de resiliência e esperança. Esse sentimento ajuda a superar a ansiedade gerada pela descoberta da doença, sua vivência e a preocupação com a morte. A maternidade pode atuar como um fator positivo, incentivando as mulheres a continuarem o tratamento antirretroviral, devido ao desejo de verem seus filhos crescerem e participarem de sua educação^(12)^.

A escolaridade é um dado importante para analisar a adesão e eficácia do tratamento, pois há uma relação direta entre o nível de escolaridade e a capacidade de acesso e compreensão das informações relacionadas ao HIV/aids. Além disso, um maior grau de escolaridade pode aumentar as chances de melhores oportunidades de emprego, tanto no aspecto financeiro quanto mental. Por outro lado, um baixo nível de escolaridade está associado à dificuldade de compreender o que é o vírus, a importância da TARV e suas implicações na vida^(20,21)^.

No que se refere à profissão, a maioria das entrevistadas relatou estar desempregada. Para a pesquisa, a profissão influencia diretamente no aspecto financeiro. Historicamente, as mulheres fazem parte da população que trabalhava com serviços domésticos, seja em suas próprias casas ou cuidando de outras famílias. Embora esse cenário esteja mudando, ainda se encontram muitos desses casos no Brasil. A mulher dedicada exclusivamente ao lar pode sofrer com uma possível dependência financeira do marido ou da família, o que aumenta sua vulnerabilidade à violência, bem como os impactos em sua saúde mental e física. O estigma associado ao HIV pode resultar em situações de exclusão do mercado de trabalho, ocasionando impactos negativos na renda individual e/ou familiar^(17)^.

No que concerne aos dados relacionados ao agravo, foram analisadas informações sobre o ano do diagnóstico, a forma de transmissão e se as participantes faziam acompanhamento com psicólogo ou psiquiatra, com ou sem uso de medicação.

Nesse contexto, observou-se que a maioria das participantes foi diagnosticada entre os anos de 2016 e 2021, sendo a forma de transmissão mais frequente a via sexual. Esses dados corroboram com o boletim epidemiológico, que, ao analisar os anos de 2007 a 2022, mostrou que o período entre 2016 e 2021 apresentou o maior número de casos de HIV notificados no Sistema de Informação de Agravos de Notificação (SINAN) para a população feminina e masculina. A forma de transmissão é um dado importante neste estudo, pois permite compreender possíveis falhas na promoção e adesão ao uso de métodos de prevenção de barreira, especialmente entre a parcela da população que acredita não precisar utilizar esses métodos por se considerar fora do “grupo de risco”, como as mulheres casadas^(12)^.

No que se refere ao acompanhamento por psicólogo ou psiquiatra, a maioria das mulheres confirmou receber esse tipo de suporte. A saúde mental é de suma importância no cotidiano das mulheres que vivem com HIV, já que conviver com uma doença sem cura, que requer medicação contínua, além das incertezas relacionadas às vulnerabilidades do dia a dia, pode desencadear sintomas como ansiedade e depressão. O acompanhamento psicológico auxilia no reconhecimento de sentimentos como o ódio de si mesma ou do parceiro que causou a exposição, a culpa por não ter se prevenido, a percepção negativa da doença, o pânico diante da morte e pensamentos sobre a impossibilidade de cura, fatores que podem afetar a saúde mental e levar ao aparecimento de sintomas depressivos e ansiosos^(21)^.

Quando questionadas sobre o uso de medicações psicotrópicas, sete das oito entrevistadas confirmaram o uso diário desses medicamentos. Pacientes com HIV e problemas de saúde mental requerem mais atenção e cuidado ao aderirem à terapia antirretroviral, pois a utilização de medicação psiquiátrica está associada, de maneira negativa, à adesão à terapia antirretroviral, devido aos estigmas e à influência de mais fármacos no organismo. Isso se deve ao fato de que a introdução de mais um regime medicamentoso, além dos antirretrovirais, evidencia a necessidade de uma rotina adicional de medicamentos, exigindo a análise de possíveis interações entre os fármacos, além dos próprios efeitos colaterais que os medicamentos psiquiátricos podem ocasionar. Esses fatores podem influenciar a autopercepção do indivíduo e sua adesão à TARV^(21,22)^.

### Os sentimentos frente à descoberta da doença e a relação com o tratamento

O presente estudo constatou que a população feminina apresenta vulnerabilidades em diversos âmbitos, e a exposição ao HIV não é exceção. Além do descaso em relação a esse público, também ocorrem subnotificações de casos, o que contribui para o aumento da incidência do HIV nesse segmento populacional^(10,12)^.

Com o surgimento da epidemia de HIV no século XX, houve a delimitação de um “grupo de risco” para a aquisição do vírus, o que gerou preconceitos em relação a esse público. De acordo com a crença popular, a doença só afetaria pessoas com orientação sexual homossexual, comportamentos promíscuos e pertencentes à classe média alta^(20)^.

Como resultado, as PVHIV passaram a sofrer autoculpabilização, enfrentando diferentes processos de aceitação diante do diagnóstico. As dificuldades e a demora da medicina em reconhecer o público feminino como vulnerável à infecção facilitaram o crescimento silencioso do HIV/aids, reforçando desigualdades de gênero. A partir das falas presentes na pesquisa, destacam-se as incertezas e o sentimento de desespero diante da descoberta de uma doença que carrega tanto preconceito, somado ao sofrimento causado pelo julgamento moral, inclusive por parte de profissionais, o abandono do companheiro e até mesmo o surgimento de pensamentos suicidas^(19)^.

O diagnóstico da infecção pelo HIV é considerado um evento profundamente negativo na vida das pessoas, pois é associado a uma doença estigmatizante na sociedade, afetando a dinâmica familiar e social, e causando isolamento e, muitas vezes, abandono por parte de si e dos outros^(5)^.

A Teoria Transpessoal do Cuidado Humano, de Margaret Jean Watson, permite identificar nas declarações das mulheres a necessidade de aplicar o cuidado de forma integral, reconhecendo que o ser humano é dotado de emoções, com crises pessoais e externas, e com diferentes níveis de dificuldade em relação à aceitação da doença^(12,15)^.

Verifica-se que a adesão ao tratamento é imprescindível para o controle do vírus no organismo, evitando a progressão para a fase de aids e reduzindo as chances de infecções oportunistas. A adesão ao tratamento é fundamental para que a carga viral se torne indetectável, com menos de 40 cópias/mL do vírus no sangue, sendo necessário que o esquema terapêutico atinja uma taxa de adesão igual ou superior a 95% das doses prescritas^(1)^.

### Impacto na saúde mental de mulheres vivendo com HIV/aids e sua relação com o profissional de saúde

A pessoa vivendo com HIV, sem o devido auxílio, pode desenvolver doenças psiquiátricas ou apresentar piora em condições preexistentes, como depressão e ansiedade patológica. Isso pode prejudicar a adesão ao tratamento de várias formas, dificultando o processo de melhoria da qualidade de vida e o aumento da taxa de sobrevida^(21)^.

Pelas respostas das entrevistadas, é evidente que sintomas como ansiedade e depressão afetam o cotidiano das pacientes, prejudicando a adesão à medicação e, em alguns casos, levando à desistência parcial ou total do tratamento. Além disso, o medo da morte é uma forma extrema de ansiedade, que impacta profundamente a vida e as decisões das pessoas. Por isso, esses sintomas devem ser reconhecidos a tempo para que o acompanhamento adequado seja iniciado, permitindo que a pessoa vivendo com HIV/aids seja devidamente assistida em seu tratamento, sem desistir ou postergá-lo, o que agravaria ainda mais a doença^(8)^.

Quando os profissionais de saúde ignoram esses sintomas, podem aumentar a possibilidade de piora no quadro clínico, uma vez que a pessoa vivendo com HIV/aids pode se sentir negligenciada em seus aspectos biopsicossociais^(22)^. Minimizar esses sentimentos contribui para o isolamento, a baixa autoestima, a solidão, a ansiedade, a depressão e os baixos níveis de esperança, podendo levar ao abandono do tratamento. Assim, é notável que uma boa qualidade de vida pode ser o diferencial na aceitação e na conduta pessoal do paciente^(8,21)^.

Em qualquer terapia, o início do tratamento é o período mais difícil para a adesão, devido à adaptação à nova rotina diária de medicação e aos efeitos colaterais. As PVHIV desenvolvem mecanismos de resiliência para continuar o tratamento de forma consistente^(11)^.

O profissional de saúde deve reforçar e apoiar o sistema de crenças como um dos mecanismos de controle emocional, facilitando a formação de vínculo e compreensão entre paciente e profissional. Dentro da perspectiva da Teoria Transpessoal do Cuidado Humano, pode-se inferir que as práticas de cuidado devem permitir o desenvolvimento de ações de promoção e a aceitação da expressão de sentimentos, tanto positivos quanto negativos, incentivando um sistema de valores humanísticos e altruístas^(21)^.

A longo prazo, a dificuldade do paciente em lidar com sua doença acarreta prejuízos ao próprio tratamento. Esse dano inibe a capacidade do paciente de enfrentar situações estressantes adicionais causadas pelo diagnóstico de HIV. Assim, a ideia principal para definir o conceito de adesão é a forma como a pessoa que vive com HIV/aids responderá às recomendações acordadas com o profissional de saúde e como se dará o processo de adaptação à rotina de um determinado regime terapêutico no cotidiano^(1,11)^.

Analisando as falas das entrevistadas, percebe-se a necessidade de praticar a comunicação, realizar uma escuta qualificada e despir-se de julgamentos. Dessa forma, o profissional deve desenvolver uma relação que fomente um vínculo profundo de confiança e compreensão, favorecendo a adesão à medicação ao entender os motivos da possível recusa e atuar de maneira incisiva sobre eles. Assim, por meio da análise da situação e das dificuldades expostas pelo paciente através do diálogo, o profissional deve observar suas necessidades, ouvir suas posições, identificar problemas e planejar ações em conjunto para o bem-estar geral, dentro da realidade em que o paciente vive^(21,22)^.

### Limitações do estudo

As limitações do estudo estão relacionadas à pandemia de COVID-19, que afetou os meses de coleta de dados, reduzindo a quantidade de profissionais disponíveis e o número de pacientes atendidos. Para concluir a coleta de dados, a pesquisadora adotou estratégias como o uso de equipamentos de proteção individual (EPIs) e o agendamento das entrevistas em dias e horários combinados com as mulheres.

### Contribuições para a área da Enfermagem

O estudo contribui de maneira significativa para o cuidado com a saúde mental das mulheres vivendo com HIV/aids, ressaltando a importância de uma assistência empática para a adesão à terapia antirretroviral. A abordagem deve garantir que todas as pacientes possam usufruir dos benefícios do tratamento sem se exporem ao estigma que recai sobre as PVHIV. Além disso, o estudo propõe um atendimento humanizado, que promova estratégias de acompanhamento das vulnerabilidades e melhore a assistência prestada.

## CONSIDERAÇÕES FINAIS

Com base nos resultados deste estudo, conclui-se que as mulheres vivendo com HIV enfrentam um momento de grande fragilidade, tornando-se ainda mais vulneráveis durante o processo de descoberta da infecção, principalmente devido ao impacto que o vírus causa em sua dinâmica individual e coletiva. A ansiedade diante da morte e o medo do preconceito da família, da sociedade e até mesmo dos profissionais de saúde geram sentimentos de desespero, abandono e, em alguns casos, a negação da doença.

Além disso, o estudo revela que a saúde mental tem uma influência considerável na adesão aos medicamentos antirretrovirais. A descoberta de uma doença crônica, incurável e estigmatizada na sociedade pode ser um fator desencadeador de problemas psíquicos ou um agravante para condições preexistentes, como ansiedade e depressão. Dessa forma, a ansiedade patológica e a depressão são sintomas que refletem nas ações cotidianas das pacientes, afetando a adesão à rotina medicamentosa. Isso pode influenciar negativamente a carga viral, levando ao surgimento de doenças oportunistas, à progressão para aids e até mesmo à morte.
